# Complementary Effects of Adaptation and Gain Control on Sound Encoding in Primary Auditory Cortex

**DOI:** 10.1523/ENEURO.0205-20.2020

**Published:** 2020-11-12

**Authors:** Jacob R. Pennington, Stephen V. David

**Affiliations:** 1 Department of Mathematics, Washington State University, Vancouver, WA, 98686; 2 Department of Otolaryngology, Oregon Health and Science University, Portland, OR, 97239

**Keywords:** auditory encoding, computational modeling, gain control, sensory context, synaptic adaptation

## Abstract

An important step toward understanding how the brain represents complex natural sounds is to develop accurate models of auditory coding by single neurons. A commonly used model is the linear-nonlinear spectro-temporal receptive field (STRF; LN model). The LN model accounts for many features of auditory tuning, but it cannot account for long-lasting effects of sensory context on sound-evoked activity. Two mechanisms that may support these contextual effects are short-term plasticity (STP) and contrast-dependent gain control (GC), which have inspired expanded versions of the LN model. Both models improve performance over the LN model, but they have never been compared directly. Thus, it is unclear whether they account for distinct processes or describe one phenomenon in different ways. To address this question, we recorded activity of neurons in primary auditory cortex (A1) of awake ferrets during presentation of natural sounds. We then fit models incorporating one nonlinear mechanism (GC or STP) or both (GC+STP) using this single dataset, and measured the correlation between the models’ predictions and the recorded neural activity. Both the STP and GC models performed significantly better than the LN model, but the GC+STP model outperformed both individual models. We also quantified the equivalence of STP and GC model predictions and found only modest similarity. Consistent results were observed for a dataset collected in clean and noisy acoustic contexts. These results establish general methods for evaluating the equivalence of arbitrarily complex encoding models and suggest that the STP and GC models describe complementary processes in the auditory system.

## Significance Statement

Computational models are used widely to study neural sensory coding. However, models developed in separate studies are often difficult to compare because of differences in stimuli and experimental preparation. This study develops an approach for making systematic comparisons between models that measures the net benefit of incorporating additional nonlinear elements into models of auditory encoding. This approach was then used to compare two different hypotheses for how sensory context, that is, slow changes in the statistics of the acoustic environment, influences activity in auditory cortex. Both models accounted for complementary aspects of the neural response, indicating that a hybrid model incorporating elements of both models provides the most complete characterization of auditory processing.

## Introduction

The sound-evoked spiking activity of an auditory neuron can be modeled as a function of a time-varying stimulus plus some amount of error, reflecting activity that cannot be explained by the model. Prediction error can reflect experimental noise (physiological noise or recording artifacts), but in many cases it also reflects a failure of the model to account for some aspects of sound-evoked activity (Sahani and Linden, 2003b). A common encoding model used to study the auditory system is the linear-nonlinear spectro-temporal receptive field (STRF), or LN model (David et al., 2007; Calabrese et al., 2011; Rahman et al., 2019). According to the LN model, the time-varying response of a neuron can be predicted by convolution of a linear filter with the sound spectrogram followed by a static rectifying nonlinearity. The LN model is a generalization of the classical STRF, which does not specify a static nonlinearity but provides a similar description of auditory coding (Aertsen and Johannesma, 1981; Eggermont et al., 1983; deCharms et al., 1998; Klein et al., 2000; Theunissen et al., 2001). The LN model has been useful because of its generality; that is, because it provides a representation of a neuron’s response properties that holds for arbitrary stimuli (Aertsen and Johannesma, 1981; Theunissen et al., 2000).

Despite the relative value of the LN model, it fails to account for important aspects of auditory coding, particularly in more central auditory areas such as primary auditory cortex (A1; Machens et al., 2004; David et al., 2007; Atencio and Schreiner, 2008). Several studies have identified nonlinear selectivity for spectro-temporal sound features (Eggermont, 1993; Atencio et al., 2008; Sadagopan and Wang, 2009; Kozlov and Gentner, 2016). In addition, auditory neurons undergo slower changes in response properties that reflect the sensory context (Hiroki and Zador, 2009; Rabinowitz et al., 2012). A canonical example of context-dependent changes is stimulus-specific adaptation (SSA), where responses are reduced for repeating versus oddball tones (Ulanovsky et al., 2003). Context-dependent changes in coding are also apparent in responses to clean (undistorted) versus noisy stimuli (Moore et al., 2013; Rabinowitz et al., 2013; Mesgarani et al., 2014). These context effects can last hundreds of milliseconds and reflect nonlinear computations outside of the scope of a linear filter (David and Shamma, 2013). Several studies have attempted to bridge this gap in model performance by extending the LN model to incorporate experimentally-observed nonlinearities, including short-term synaptic plasticity (STP) and contrast-dependent gain control (GC; Rabinowitz et al., 2012; Cooke et al., 2018; Lopez Espejo et al., 2019). These models show improved performance over the LN model and point to mechanisms that explain contextual effects.

The improved performance of these alternatives to the LN model is well established, but the extent to which they describe distinct, complementary mechanisms within the brain is not clear. It has been suggested, for example, that STP may in fact contribute to GC (Carandini et al., 2002; Rabinowitz et al., 2011). At the same time, both gain control and STP have been implicated in the robust coding of natural stimuli in noise (Rabinowitz et al., 2013; Mesgarani et al., 2014).

The complementarity of these effects has been difficult to establish because thus far they have been tested on datasets recorded from different experimental preparations and using different stimulus sets (Rabinowitz et al., 2012; David and Shamma, 2013; Lopez Espejo et al., 2019). To address this issue, we tested both STP and GC models on two natural sounds datasets, collected from A1 of unanesthetized ferret. The first was comprised of a large collection of diverse natural sounds, while the second contained only ferret vocalizations with and without additive broadband noise. We focused on natural sound coding because LN models are limited in their ability to predict responses to natural sounds in A1 (Theunissen et al., 2000; David et al., 2009; Sharpee et al., 2011).

With the models on equal footing, we compared their performance to each other and to a standard LN model. Both models showed improved performance over the LN model, but a model combining the STP and GC mechanisms performed better than either one alone. Additionally, we found a low degree of similarity between the STP and GC models’ predictions after accounting for the LN model’s contributions. These results suggest that models for STP and GC are not equivalent, and in fact account for complementary components of auditory cortical coding.

## Materials and Methods

### Experimental procedures

#### Data collection

All procedures were approved by the Oregon Health and Science University Institutional Animal Care and Use Committee (protocol IP00001561) and conform to standards of the Association for Assessment and Accreditation of Laboratory Animal Care (AAALAC).

Before experiments, all animals (*Mustela putorius furo*, seven males) were implanted with a custom steel head post to allow for stable recording. While under anesthesia (ketamine followed by isoflurane) and under sterile conditions, the skin and muscles on the top of the head were retracted from the central 4-cm diameter of skull. Several stainless-steel bone screws (Synthes, 6 mm) were attached to the skull, the head post was glued on the mid-line (3 M. Durelon), and the site was covered with bone cement (Zimmer Palacos). After surgery, the skin around the implant was allowed to heal. Analgesics and antibiotics were administered under veterinary supervision until recovery.

After animals fully recovered from surgery and were habituated to a head-fixed posture, a small craniotomy (1–2 mm in diameter) was opened over A1. Neurophysiological activity was recorded using tungsten microelectrodes (1–5 MO, A.M. Systems). One to four electrodes positioned by independent microdrives (Alpha-Omega Engineering EPS) were inserted into the cortex.

Electrophysiological activity was amplified (A.M. Systems 3600), digitized (National Instruments PCI-6259), and recorded using the MANTA open-source data acquisition software (Englitz et al., 2013). Recording site locations were confirmed as being in A1 based on tonotopy, relatively well-defined frequency tuning and short response latency (Kowalski et al., 1996).

Spiking events were extracted from the continuous raw electrophysiological trace by principal components analysis and k-means clustering (David et al., 2009). Single unit isolation was quantified from cluster variance and overlap as the fraction of spikes that were likely to be from a single cell rather than from another cell. Only units with >80% isolation were used for analysis.

Stimulus presentation was controlled by custom software written in MATLAB (version 2012A, MathWorks). Digital acoustic signals were transformed to analog (National Instruments PCI6259) and amplified (Crown D-75a) to the desired sound level. Stimuli were presented through a flat-gain, free-field speaker (Manger) 80 cm distant, 0° elevation and 30° azimuth contralateral to the neurophysiological recording site. Before experiments, sound level was calibrated to a standard reference (Brüel & Kjær). Stimuli were presented at 60–65 dB SPL.

#### Natural stimuli

The majority of data included in this study were collected during presentation of a library of natural sounds (set 1: 93, 3 s/sample, set 2: 306, 4 s/sample). Some of these sounds (set 1: 30%, set 2: 10%) were ferret vocalizations. The vocalizations were recorded in a sound-attenuating chamber using a commercial digital recorder (44-kHz sampling, Tascam DR-400). Recordings included infant calls (one week to one month of age), adult aggression calls, and adult play calls. No animals that produced the vocalizations in the stimulus library were used in the current study. The remaining natural sounds were drawn from a large library of human speech, music and environmental noises developed to characterize natural sound statistics (McDermott et al., 2013).

Neural activity was recorded during three repetitions of these stimuli (set 1: 90, set 2: 288) in random order and either 24 or 30 repetitions of the remaining stimuli (set 1: 3, set 2: 18), all ferret vocalizations, presented on random interleaved trials with 1–3 s of silence between stimuli. The low-repetition data were used for model estimation and the high-repetition data were used for model validation.

A second dataset was collected during presentation of ferret vocalizations in clean and noisy conditions; 43-s vocalizations were each presented without distortion (clean) and with additive Gaussian white noise [0 dB signal-to-noise ratio (SNR), peak-to-peak]. The noise started 0.5 s before the onset and ended 0.5 s following the offset of each vocalization. A distinct frozen noise sample was paired with each vocalization to allow repetition of identical noisy stimuli. Stimuli were presented at 65 dB SPL with 1-s interstimulus interval.

### Modeling framework

#### Cochlear filterbank

To represent the input for all the encoding models, stimulus waveforms were converted into spectrograms using a second-order gammatone filterbank (Katsiamis et al., 2007). The filterbank included *F *=* *18 filters with *f_i_* spaced logarithmically from *f_low_* = 200 to *f_high_* = 20,000 Hz. After filtering, the signal was smoothed and down-sampled to 100 Hz to match the temporal bin size of the peristimulus time histogram (PSTH), and log compression was applied to account for the action of the cochlea.

#### LN model

The first stage of the LN model applied a finite impulse response (FIR) filter, *h*, to the stimulus spectrogram, *s*, to generate a linear firing rate prediction (*y_lin_*):

(1) $$y_{lin}(t)=\sum_{f}^{F}\sum_{u}^{U}h_{f,u};s(f,t-u).$$

For this study, the filter consisted of *F *=* *18 spectral channels and *U *=* *15 temporal bins (10 ms each). In principle, this step can be applied to the spectrogram as a single 18 × 15 filter. In practice, the filter was applied in two stages: multiplication by an 18 × 3 spectral weighting matrix followed by convolution with a 3 × 15 temporal filter. Previous work has shown that this rank-3 approximation of the full filter is advantageous for prediction accuracy in A1 (Thorson et al., 2015).

The output of the filtering operation was then used as the input to a static sigmoid nonlinearity that mimicked spike threshold and firing rate saturation to produce the final model prediction. For this study, we used a double exponential nonlinearity:

(2) $$y(t)=b\;+\;ae^{-e^{k(y_{lin}(t)-s))}},$$

where the baseline spike rate, saturated firing rate, firing threshold, and gain are represented by *b*, *a*, *s*, and *k*, respectively.

#### STP model

The output of each spectral channel served as the input to a virtual synapse that could undergo either depression or facilitation (Tsodyks et al., 1998). In this model, the number of presynaptic vesicles available for release within a virtual synapse is dictated by the fraction of vesicles released by previous stimulation, *u_i_*, and a recovery time constant, *τ_i_*. For depression, *u_i_* > 0, and the fraction of available vesicles, *d*(*t*), is updated,

$$d_{i}(t)=d_{i}(t-1)-u_{i}s_{i}(t-1)d_{i}(t-1)+\frac{1-d_{i}(t-1)}{\tau_{i}}.$$

For facilitation, *u_i_* < 0, and *d*(*t*) is updated,

$$d_{i}(t)=d_{i}(t-1)-u_{i}s_{i}(t-1)[2-d_{i}(t-1)]+\frac{1-d_{i}(t-1)}{\tau_{i}}.$$

The input to the *i*-th synapse, *s_i_*, is scaled by the fraction of available vesicles, *d_i_*:

(3) $$s_{d_{i}}(t)=d_{i}(t)s_{i}(t).$$

The scaled output, $s_{d_{i}}(t)$, is then used as the input to a temporal filter, identical to the one used in the LN model. Three virtual synapses were used in this study to match the rank-3 STRF approximation, for a total of six free parameters *τ_i_*, *u_i_*, *i *=* *0, 1, 2. Values of *τ* and *u* reported in the results represent the mean across all three virtual synapses.

#### GC model

The GC model was adapted from Rabinowitz et al. (2012). In this model, the parameters of the output nonlinearity depend on a linear weighted sum of the time-varying stimulus contrast. For each stimulus, the contrast, *C*, within a frequency band, *f*, was calculated as the coefficient of variation,

(4) $$C_{f}(t)=\frac{\sigma_{f}(t)}{\mu_{f}(t)},$$

within a 70-ms rolling window offset by 20 ms. *σ_f_* is the SD and *μ_f_* is the mean level within that window (dB SPL). In the GC model’s original formulation, a linear filter with fittable coefficients would then be applied to the stimulus contrast (Rabinowitz et al., 2012). For this study, we found that a simple contrast weighting provided more accurate predictions. Our implementation used a fixed filter that summed stimulus contrast across frequencies and at a single time point. Thus, the contrast at each point reflected the ratio in Equation 4 computed over the window 20–90 ms preceding the current moment in time. The output, *K*, of this summation was then used to determine the parameters of the output nonlinearity in (2) such that the *i*-th parameter, *θ_i_*, was determined from the base value, $\theta_{i_{0}}$, that would normally be fitted in (2) and a contrast weighting term, $\theta_{i_{1}}$:

(5) $$K(t)=\sum_{f}^{F}C_{f}(t)$$

(6) $$\theta_{i}(t)=\theta_{i_{0}}+(\theta_{i_{1}}-\theta_{i_{0}})K(t).$$

With this formula, it is necessary to know both $\theta_{i_{1}}$ and $\theta_{i_{0}}$ to determine the impact of contrast on any particular parameter. However, we did not find any significant differences in the base values $\theta_{i_{0}}$ between improved and non-improved cells. In the results we instead report the difference, $\left(\theta_{i_{1}}-\theta_{i_{0}}\right)$, which represents the slope of the linear relationship proposed by the model.

#### Model optimization

Models were optimized using the L-BFGS-B gradient descent algorithm implemented in the SciPy Python library (Virtanen et al., 2020). This optimization minimized the mean-squared error (MSE) between a neuron’s time-varying response, averaged across any repeated presentations of the same stimulus, and the model’s prediction. Post-fitting performance was evaluated based on the correlation coefficient (Pearson’s *R*) between prediction and response, adjusted for the finite sampling of validation data (Hsu et al., 2004).

Because of the difficult nonlinear optimization problem posed by the models used in this study, we were not able to reliably fit all model parameters simultaneously. Instead, when optimizing the GC+STP model, it was necessary to fit the STP model parameters before fitting the GC model parameters. We began by fitting only the linear STRF portion of the model, using coarse stopping criteria, with the other portions of the model excluded. Next, we incorporated and optimized the STP and static output nonlinearity parameters while keeping the STRF parameters fixed. This was followed by simultaneous optimization of all LN and STP parameters using finer stopping criteria. Next, the GC and STP parameters were optimized while keeping LN parameters fixed. Finally, all model parameters were optimized simultaneously. Other model fits followed the same process, but without the additional parameters where appropriate. Compared with optimizing all parameters in a single step we found that, on average, using this heuristic approach reduced overfitting and avoided more local minima. However, we emphasize that we were not able to remedy these issues entirely, as is typical for encoding models of complex spiking data.

#### Equivalence analysis

Equivalence of STP and GC models was quantified using the partial correlation between the time-varying response to the validation stimuli predicted by each model, computed relative to the prediction by the LN model (Baba et al., 2004). If the two models were equivalent and deviated from the LN model in exactly the same way, the partial correlation would be 1.0. If they deviated in completely different ways, the partial correlation would be 0.0.

Because dataset size was finite, noise in the estimation data produced uncertainty in model parameter estimates, which biased partial correlation values to be less than 1. To compute an upper bound on equivalence, we split the estimation dataset in half and fit the same model (STP or GC) to both halves. We then measured equivalence between two fits of the same model (*W_half_*), one for each half of the estimation data, using the models’ predictions for the full validation dataset.

The resulting within-model equivalence scores were corrected to account for noisier model estimates that resulted from using half as much data to fit each model as in the main analysis. Three measurements were used for the correction: within-model equivalence, *W_half_*, computed as described above; between-model equivalence using the full dataset, *B_full_*; and between-model equivalence using opposite halves of the estimation data, *B_half_*, similar to the within-model computation. Corrected within-model equivalence, *W*_+_, was computed for each of the STP and GC models as:

(7) $$W_{+}=\left(\frac{B_{full}}{B_{half}}\right)W_{half}\cdot$$

The ratio $\frac{B_{full}}{B_{half}}$ represents the increase in equivalence expected if the within-model comparison could be performed on the full dataset. The values for within-model equivalence reported in the results are these corrected scores.

We also estimated an upper bound for the comparison of relative improvements to prediction correlation. As with the partial correlation computations, relative performance for the full validation dataset was compared within-model by using separate halves of the estimation dataset for fitting.

#### Reliability metric

Neurons with noisy, unreliable responses necessarily have less reliable model fits, which in turn are biased toward low equivalence values. To quantify the reliability of neural responses, we used a variation on the SNR, based on methods developed by Sahani and Linden (Sahani and Linden, 2003a,b). A typical SNR definition would be:

(8) $$SNR=\frac{Signal\;Power}{Noise\;Power}=\frac{Signal\;Power}{Total\;Power-Signal\;Power}.$$

However, SNR values can be difficult to compute for sparse spiking data. In an extreme case, noise power would be zero for a train of zero spikes, leading to indeterminate SNR. To avoid this problem, we measured response reliability as

(9) $$Reliability=\frac{Signal\;Power}{Total\;Power}.$$

Since total power is the sum of signal and noise power, this approach avoids the issue of sparse responses in the noise computation. Interpretation of the metric is also straightforward. For example, a reliability of 0.5 indicates a response that is 50% signal and 50% noise. For each neuron and stimulus, we measured signal and total power for the spiking response *y_j_* to the *j*-th stimulus repetition as:

(10) $$\begin{array}{ll} {\left(Total\;Power\right)}_{j} & =〈y_{j},y_{j}〉\\ {\left(Signal\;Power\right)}_{j} & =\frac{1}{m}\sum_{k=1}^{m}〈y_{j},y_{k}〉,\;\;j\neq k \end{array},$$

where 〈,〉 denotes a dot product and *m* is the total number of repetitions. This process was repeated for each repetition of each stimulus to obtain a reliability measure for the *i*-th stimulus:

(11) $${\left(Reliability\right)}_{i}=\frac{1}{m}\sum_{j=1}^{n}\frac{{\left(Signal\;Power\right)}_{j}}{{\left(Total\;Power\right)}_{j}}.$$

A neuron’s overall reliability was then calculated as the mean of the per-stimulus values:

(12) $$Reliability=\frac{1}{n}\sum_{i=1}^{n}{\left(Reliability\right)}_{i}.$$

#### Code accessibility

The python-based model estimation software described in the paper is freely available online at https://github.com/LBHB/NEMS and https://github.com/LBHB/nems_db. Analyses for this study were run on an in-house compute cluster using Intel core-i7 CPUs running the Ubuntu Linux operating system version 16.04.

## Results

### Models for encoding of natural sounds by neurons in A1

**Figure 1.**
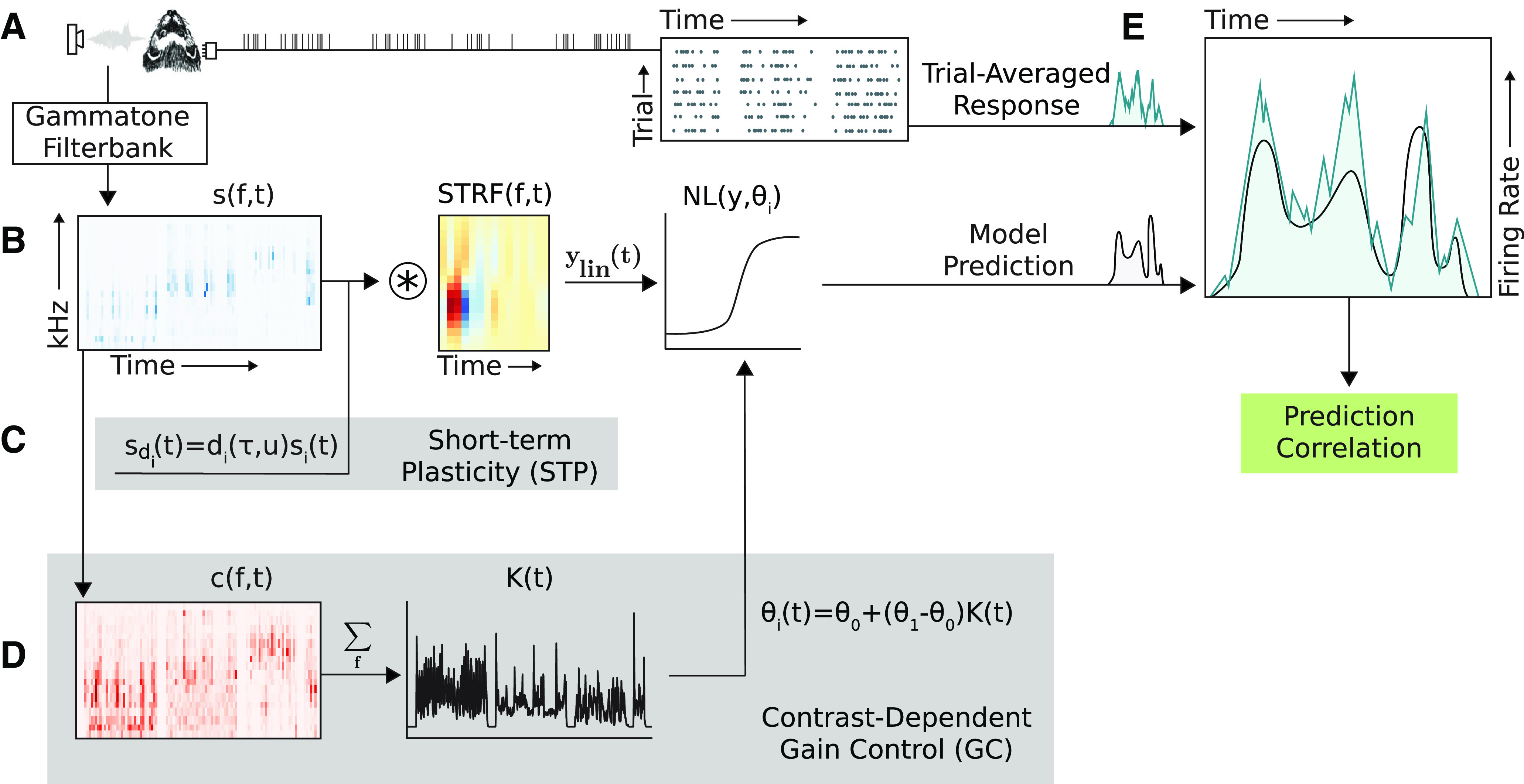
Schematic of four different model architectures for sound encoding by neurons in auditory cortex. ***A***, Single neuron activity was recorded from A1 of awake, passively listening ferrets during presentation of a large set of natural sound stimuli. The trial-averaged response to each sound was calculated as the instantaneous firing rate using 10-ms bins. Sound waveforms were transformed into 18-channel spectrograms with log-spaced frequencies for input to the models. ***B***, Linear nonlinear (LN) model: stimulus spectrogram is convolved with a linear spectro-temporal filter followed by nonlinear rectification. ***C***, Short-term plasticity (STP) model: simulated synapses depress or facilitate spectral stimulus channels before temporal convolution. ***D***, Gain control (GC) model: the coefficient of variation (contrast) of the stimulus spectrogram within a rolling window is summed across frequencies. Parameters for the nonlinear rectifier are scaled by time-varying contrast. ***E***, Model performance is measured by the correlation coefficient (Pearson’s *R*) between the trial-averaged response and the model prediction. The four architectures were defined as follows: LN, ***B*** only; STP, ***B*** and ***C***; GC, ***B*** and ***D***; GC+STP, ***B–D***.

To compare performance of the short-term plasticity (STP) and gain control (GC) models directly, we recorded the activity of *n *=* *540 neurons in A1 of awake, non-behaving ferrets during presentation of a large natural sound library (Fig. 1). We then compared how models incorporating STP or GC nonlinearities accounted for sound-evoked activity in the same dataset. See Table 1 for a list of all statistical tests used, with individual tests referenced by superscript throughout the results.

**Table 1:** Statistical tests reported in the Results, labeled in the text by the letters in the left-hand column

	Distribution	Test	Statistic	*p* value	Sample size	animals
a	Non-parametric	Permutation test	N/A	*p *<* *0.05, 468 sig.	*n *=* *540	*n *=* *7
b	Non-parametric	Wilcoxon signed-rank	*T *=* *2.17 × 10^4^	*p *=* *7.54 × 10^−30^	*n *=* *468	*n *=* *7
c	Non-parametric	Wilcoxon signed-rank	$T=2.39\times 10^{4}$	$p=3.55\times 10^{-26}$	*n *=* *468	*n *=* *7
d	Non-parametric	Wilcoxon signed-rank	$T=4.46\times 10^{4}$	$p=5.00\times 10^{-4}$	*n *=* *468	*n *=* *7
e	Non-parametric	Wilcoxon signed-rank	$T=2.60\times 10^{4}$	$p=5.10\times 10^{-23}$	*n *=* *468	*n *=* *7
f	Non-parametric	Wilcoxon signed-rank	$T=2.31\times 10^{4}$	$p=2.02\times 10^{-27}$	*n *=* *468	*n *=* *7
g	Non-parametric	Jackknife *t* test	*T* (varies)	*p *<* *0.05, 132 sig.	*n *=* *468	*n *=* *7
h	Normal (residuals)	Pearson’s correlation	*r *=* *0.18	$p=6.42\times 10^{-5}$	*n *=* *468	*n *=* *7
i	Normal (residuals)	Pearson’s correlation	*r *=* *0.31	$p=1.26\times 10^{-6}$	*n *=* *237	*n *=* *2
j	Normal (residuals)	Pearson’s correlation	*r *=* *0.50	$p=1.34\times 10^{-16}$	*n *=* *237	*n *=* *2
k	Non-parametric	Mann–Whitney *U*	$U=1.48\times 10^{4}$	$p=1.72\times 10^{-8}$	*n *=* *132, 336	*n *=* *7
l	Normal (residuals)	Pearson’s correlation	*r *=* *0.22	*p *=* *0.0103	*n *=* *132	*n *=* *7
m	Non-parametric	Wilcoxon signed-rank	$T=1.00\times 10^{3}$	$p=1.41\times 10^{-14}$	*n *=* *132	*n *=* *7
n	Non-parametric	Mann–Whitney *U*	$U=3.39\times 10^{3}$	$p=6.01\times 10^{-12}$	*n *=* *119, 118	*n *=* *2
o	Non-parametric	Mann–Whitney *U*	$U=4.85\times 10^{3}$	$p=3.91\times 10^{-5}$	*n *=* *119, 118	*n *=* *2
p	Non-parametric	Mann–Whitney *U*	$U=3.67\times 10^{3}$	$p=1.23\times 10^{-8}$	*n *=* *93, 141	*n *=* *7
q	Normal (residuals)	Pearson’s correlation	*r *=* *0.34	$p=1.20\times 10^{-7}$	*n *=* *234	*n *=* *7
r	Normal (residuals)	Pearson’s correlation	*r *=* *0.66	$p=1.18\times 10^{-16}$	*n *=* *122	*n *=* *2
s	Normal (residuals)	Pearson’s correlation	*r *=* *0.41	$p=2.61\times 10^{-6}$	*n *=* *122	*n *=* *2
t	Non-parametric	Mann–Whitney *U*	$U=1.61\times 10^{4}$	$p=3.53\times 10^{-6}$	*n *=* *132, 336	*n *=* *7
u	Non-parametric	Mann–Whitney *U*	$U=1.26\times 10^{4}$	$p=2.92\times 10^{-13}$	*n *=* *132, 336	*n *=* *7
v	Non-parametric	Mann–Whitney *U*	$U=2.72\times 10^{4}$	$p=1.22\times 10^{-4}$	*n *=* *132, 336	*n *=* *7
w	Non-parametric	Mann–Whitney *U*	$U=2.81\times 10^{4}$	$p=8.01\times 10^{-6}$	*n *=* *132, 336	*n *=* *7
x	Non-parametric	Mann–Whitney *U*	$U=1.56\times 10^{4}$	$p=5.90\times 10^{-7}$	*n *=* *132, 336	*n *=* *7
y	Non-parametric	Mann–Whitney *U*	$U=2.24\times 10^{4}$	$p=0.852\times 10^{-4}$	*n *=* *132, 336	*n *=* *7
z	Non-parametric	Mann–Whitney *U*	$U=1.94\times 10^{3}$	p* = 1.00	*n *=* *132, 336	*n *=* *7
aa	Non-parametric	Mann–Whitney *U*	$U=5.19\times 10^{2}$	p* = 1.00	*n *=* *132, 336	*n *=* *7
bb	Non-parametric	Mann–Whitney *U*	$U=5.28\times 10^{2}$	p* = 1.00	*n *=* *132, 336	*n *=* *7
cc	Non-parametric	Mann–Whitney *U*	$U=3.39\times 10^{3}$	p* = 1.00	*n *=* *132, 336	*n *=* *7
dd	Non-parametric	Mann–Whitney *U*	$U=1.09\times 10^{4}$	p* = 1.00	*n *=* *132, 336	*n *=* *7
ee	Non-parametric	Mann–Whitney *U*	$U=9.49\times 10^{3}$	p* = 1.00	*n *=* *132, 336	*n *=* *7
ff	Non-parametric	Mann–Whitney *U*	$U=1.86\times 10^{3}$	p* = 1.00	*n *=* *132, 336	*n *=* *7
gg	Non-parametric	Mann–Whitney *U*	$U=5.47\times 10^{2}$	p* = 1.00	*n *=* *132, 336	*n *=* *7
hh	Non-parametric	Mann–Whitney *U*	$U=2.37\times 10^{4}$	p* = 1.00	*n *=* *132, 336	*n *=* *7
ii	Non-parametric	Mann–Whitney *U*	$U=4.78\times 10^{2}$	p* = 1.00	*n *=* *132, 336	*n *=* *7
jj	Non-parametric	Mann–Whitney *U*	$U=1.40\times 10^{4}$	$p^{*}=1.68\times 10^{-3}$	*n *=* *132, 336	*n *=* *7
kk	Non-parametric	Mann–Whitney *U*	$U=1.19\times 10^{4}$	$p^{*}=2.87\times 10^{-3}$	*n *=* *132, 336	*n *=* *7
ll	Non-parametric	Wilcoxon signed-rank	$T=3.67\times 10^{3}$	$p=5.84\times 10^{-3}$	*n *=* *141	*n *=* *6
mm	Non-parametric	Wilcoxon signed-rank	$T=2.26\times 10^{3}$	$p=1.70\times 10^{-8}$	*n *=* *141	*n *=* *6
nn	Non-parametric	Wilcoxon signed-rank	$T=2.14\times 10^{3}$	$p=3.69\times 10^{-9}$	*n *=* *141	*n *=* *6
oo	Non-parametric	Wilcoxon signed-rank	$T=3.02\times 10^{3}$	$p=4.27\times 10^{-5}$	*n *=* *141	*n *=* *6
pp	Non-parametric	Wilcoxon signed-rank	$T=4.22\times 10^{3}$	*p *=* *0.105	*n *=* *141	*n *=* *6
qq	Normal (residuals)	Pearson’s Correlation	*r *=* *0.18	*p *=* *0.0345	*n *=* *141	*n *=* *6
rr	Non-parametric	Mann–Whitney *U*	*U *=* *5.02 × 10^2^	*p *=* *0.0449	*n *=* *12, 129	*n *=* *6

p* indicates Bonferroni-adjusted *p* values for 12 multiple comparisons.

We compared performance of four model architectures, fitting and evaluating each with the same dataset (Fig. 1*B–D*). The first was a standard linear-nonlinear (LN) model, which is widely used to characterize spectro-temporal sound encoding properties (Simoncelli et al., 2004; Calabrese et al., 2011; Rahman et al., 2019) and provided a baseline for the current study. The second architecture (STP model) accounted for synaptic depression or facilitation by scaling input stimuli through simulated plastic synapses (Tsodyks et al., 1998; Wehr and Zador, 2005; Lopez Espejo et al., 2019). The third (GC model) scaled a neuron’s sound-evoked spike rate as a function of recent stimulus contrast (Rabinowitz et al., 2011). A fourth architecture (GC+STP model) incorporated both STP and GC mechanisms into the LN model. The LN, STP, and GC models were implemented following previously published architectures (Rabinowitz et al., 2012; Lopez Espejo et al., 2019), and the GC+STP model combined elements from the other models in a single architecture. Data were grouped into two sets to permit unbiased model comparison: an “estimation” dataset used for fitting and a held-out “validation” dataset used for assessing model performance. The estimation dataset included a wide variety of stimuli repeated only a few times each to explore as large a portion of the stimulus space as was feasible. The validation dataset contained fewer stimuli, none of which was present in the estimation set, that were repeated many times each to obtain an accurate estimate of the time-varying firing rate.

### Complementary explanatory power by STP and gain control models

We quantified prediction accuracy using the correlation coefficient (Pearson’s *R*) between the predicted and actual PSTH response in each neuron’s validation data. Before comparing performance between models, we identified auditory-responsive neurons for which the prediction accuracy of all four models was greater than expected for a random prediction (*p *<* *0.05, permutation test, *n *=* *468/540*^a^*). Comparisons then focused on this subset. This conservative choice ensured that comparisons between model performance were not disproportionately impacted by cells for which a particular optimization failed.

**Figure 2.**
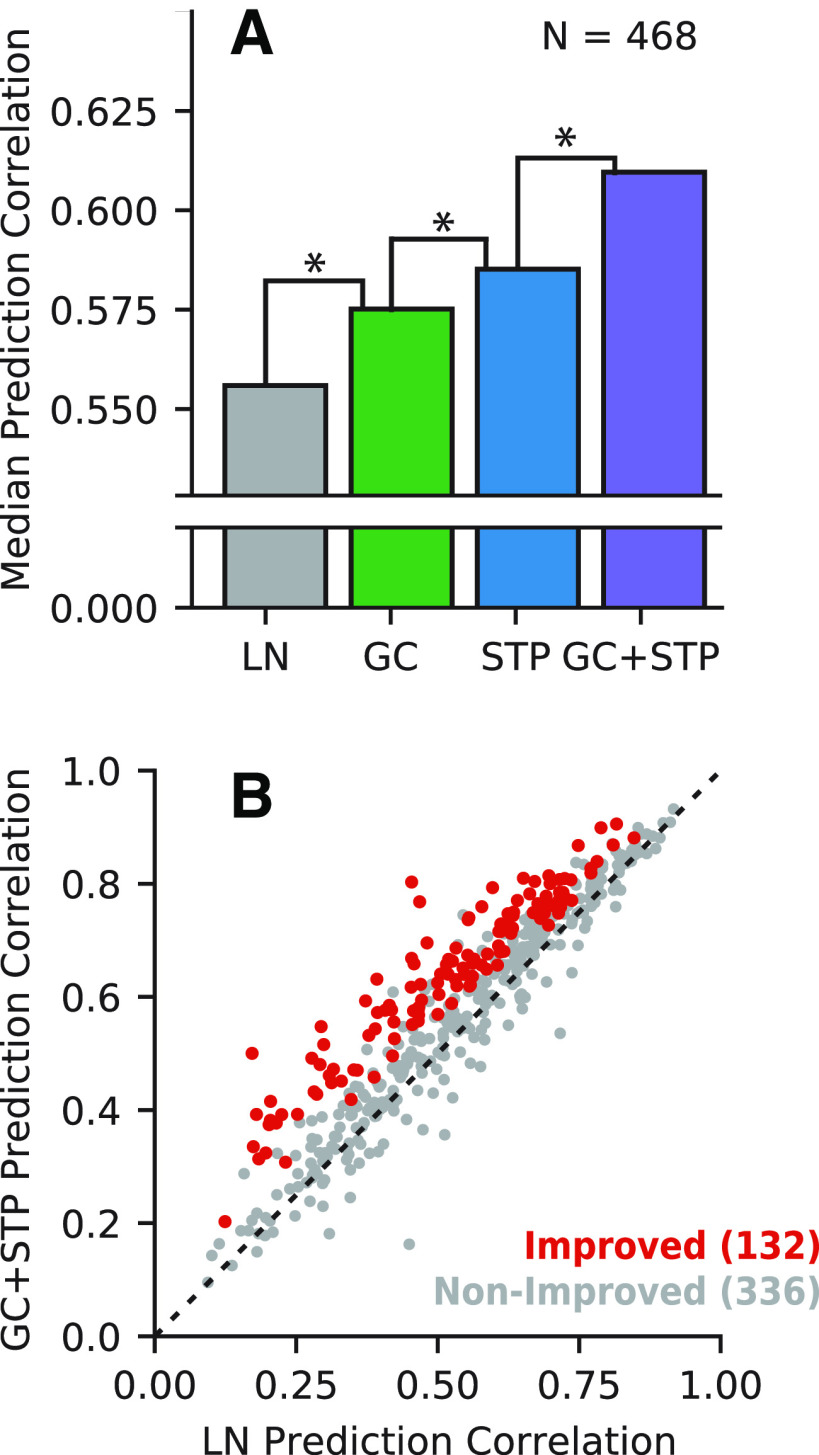
Comparison of model prediction accuracy. ***A***, Median prediction correlation for each model (*n *=* *468 neurons). Differences between LN and GC (*p =* *7.54 × 10^−30^), GC and STP (*p =* *5.00 × 10^−4^), and STP and GC+STP (*p =* *2.02 × 10^−27^) models were all significant (two-sided Wilcoxon signed-rank test). ***B***, Scatter plot compares prediction correlation by the LN model and combined GC+STP model for each neuron. Color indicates whether the combined model showed a significant improvement (red, *p *<* *0.05, permutation test) or not (gray).

A comparison of median prediction correlation across the entire set of auditory-responsive neurons (*n *=* *468; Fig. 2*A*) revealed that both the GC model and the STP model performed significantly better than the LN model (*p *=* *7.54 × 10^−30^ and *p *=* *3.55 × 10^−26^, respectively*^b^*^,^*^c^*, two-sided Wilcoxon signed-rank test), confirming previous results (Rabinowitz et al., 2012; Lopez Espejo et al., 2019). We also found that the STP model performed significantly better than the GC model (*p *=* *5.00 × 10^−24^*^d^*). If the STP and GC models were equivalent to one another, we would not expect to observe further improvement for the combined GC+STP model. Instead, we observed a significant increase in predictive power for the combined model over both the GC model and the STP model (*p *=* *5.10 × 10^–23^ and *p *=* *2.02 × 10^−27^, respectively*^e^*^,^*^f^*). The improvement for the combined model suggests that the STP and GC models describe complementary functional properties of A1 neurons.

The scatter plot in Figure 2*B* compares performance of the LN and combined models for each neuron. Among the 468 auditory-responsive neurons, 132 (28.2%) showed a significant improvement in prediction accuracy for the combined versus the LN model (*p *<* *0.05, Jackknife *t* test*^g^*). For the analyses of model equivalence and parameter distributions below, we focus on this set of improved neurons.

### Limited equivalence of STP and contrast gain model predictions

**Figure 3.**
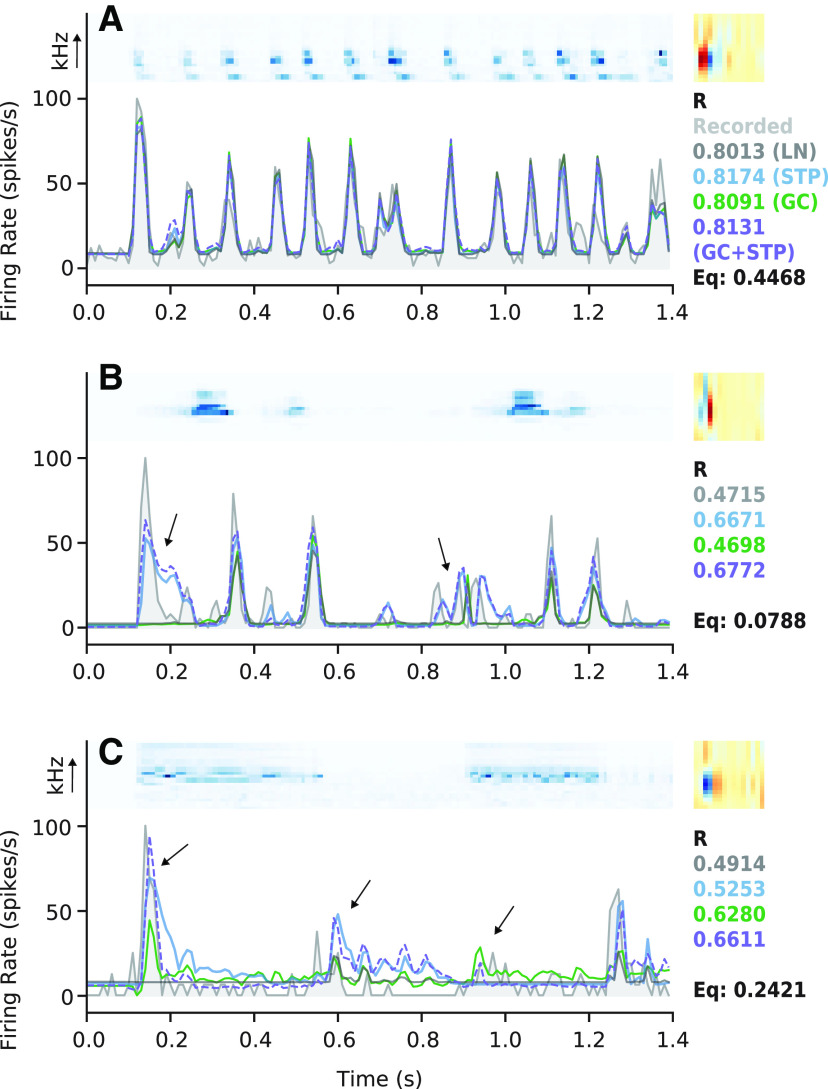
Example model fits and predictions. ***A***, Results from a neuron for which the STP and GC model predictions were not significantly better than the LN model prediction. Top left subpanel, Spectrogram from one natural sound in the validation set. Top right panel, Spectro-temporal filter from the LN model fit (right). Bottom panel, Actual PSTH response (light gray, filled) overlaid with predictions by the LN (dark gray), STP (blue), GC (green), and GC+STP (purple, dashed) models. Values at the bottom right of each panel indicate the prediction correlations for each model in the corresponding color. Below this list is the cell’s equivalence score (black, see Fig. 4*B*). The actual response was smoothed using a 30-ms boxcar filter for visualization. ***B***, Comparison for a neuron for which the STP model performed significantly better than the LN and GC models, plotted as in ***A***. Arrows indicate times for which the STP model successfully reproduced an increase in firing rate while the other models did not. The combined model prediction closely follows the STP model prediction. ***C***, Comparison for a neuron for which the GC model performed significantly better than the LN and STP models. Right-most arrow indicates a time when the GC model successfully predicted an increase in firing rate while the LN and STP models did not, while the combined model closely followed the GC model. Middle arrow indicates a time when the STP model incorrectly predicted an increase in firing rate, and the combined model nearly matched the STP model. Left-most arrow shows a time when the combined model prediction differed from both the STP and GC model predictions to more closely match a strong onset response.

A central question in this study was the extent to which the STP model’s improved performance over the LN model could be accounted for by the GC model, or vice-versa. Among non-improved cells, the two models’ predictions were often closely matched to each other and to the prediction of the LN model (Fig. 3*A*). However, for some improved neurons, the time-varying responses predicted by the STP and GC models were readily distinguishable not only from the LN model but also from each other (Fig. 3*B*,*C*). In this case, the STP and GC models both improved prediction accuracy, but they did so with low equivalence. That is, the models’ predicted responses deviated from that of the LN model in different ways. If the STP and GC models account for equivalent nonlinear properties, their predicted responses should remain similar to each other, even when differing from the LN model.

**Figure 4.**
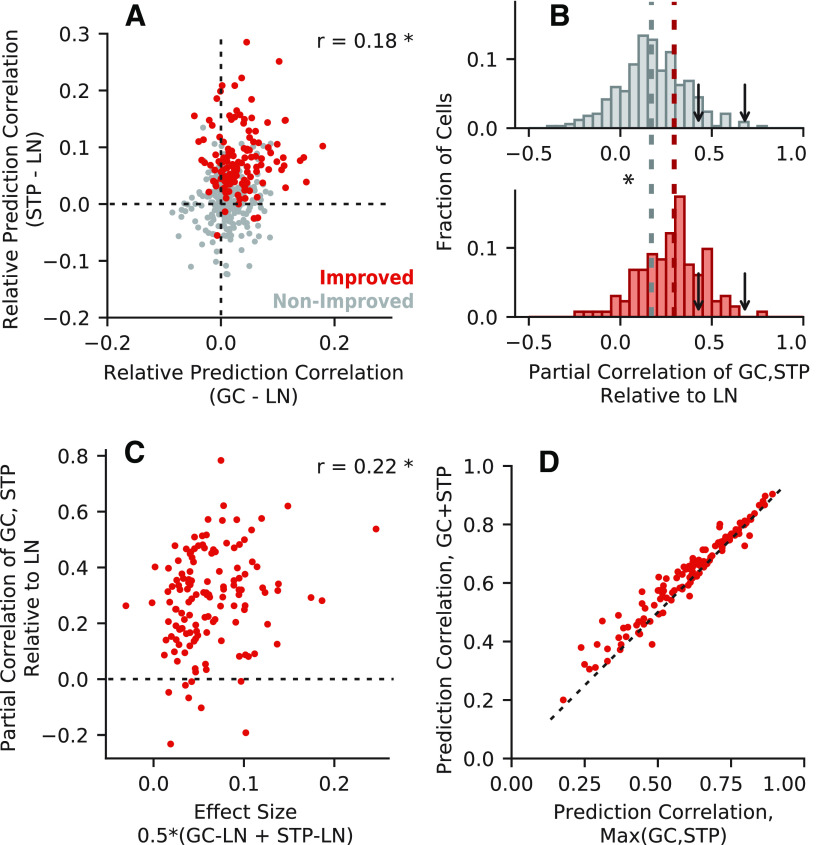
Equivalence of model predictions. ***A***, Difference in prediction correlation between the GC (horizontal axis) or STP (vertical axis) model and the LN model for each neuron (*r *=* *0.18, *p =* *6.42 × 10^−5^). Red points indicate neurons with a significant improvement for the GC+STP model over the LN model (*p *<* *0.05, permutation test); gray points indicate neurons that were not improved. ***B***, Histogram of model equivalence for each unit, measured as the partial correlation between time-varying response predicted by the STP and GC models relative to the LN model prediction. Median equivalence for improved cells (0.29, bottom, red) was significantly greater than for non-improved cells (0.17, top, gray; Mann–Whitney *U* test, *p =* *1.72 × 10^−8^). Arrows indicate median partial correlation for the GC model (0.43, left) and the STP model (0.68, right) when compared within-model, adjusted for differences in estimation data. ***C***, Scatter plot compares equivalence (vertical axis) versus effect size (horizontal axis), i.e., the average change in prediction correlation for the STP and GC models relative to the LN model, for improved cells. Only a weak relationship between equivalence and effect size was observed (*r *=* *0.22, *p =* *0.0103). ***D***, Prediction correlations for the combined GC+STP model (vertical axis) and the maximum of the GC and STP models (horizontal axis) for improved cells. Median prediction correlation was significantly higher for the combined model (0.6568) than for the greater of the individual models (0.6319; Wilcoxon signed-rank test, *p *=* *1.41 × 10^−14^).

To quantify model equivalence across neurons, we first compared the change in prediction correlation for the STP and GC models, relative to the LN model (Fig. 4*A*). If the two models were equivalent, we would expect a strong positive correlation between improvements over the LN model per neuron. However, we observed only a weak correlation (*r* = 0.18, *p *=* *6.42 × 10^−5^*^h^*).

This measure of correlation between model performance can be biased toward lower values by estimation noise that randomly impacts prediction accuracy. To control for effects of noise, we measured the same correlation statistics, but for models known to be equivalent. We performed a within-model comparison using a subset of cells for which responses to a larger collection of sounds were recorded (*n *=* *237; for details, see Materials and Methods). The within-model correlation between changes in prediction correlation was substantially larger for both the STP and GC models (*r *=* *0.50, *p *=* *1.34 × 10^−16^ and *r *=* *0.31, *p *=* *1.26 × 10^−6^, respectively*^i^*^,^*^j^*). These results indicate that model estimation noise substantially impacts measures of prediction accuracy, but equivalence of prediction correlations is still low even after accounting for estimation noise.

If two models account for the same functional properties, we also expect them to predict the same time-varying response to a validation stimulus. To test for this possibility, we measured the similarity of responses predicted by the STP and GC models. Since both models are extensions of the LN model, we discounted the contributions of the LN model to the prediction (Fig. 4*B*). We defined model equivalence for each neuron as the partial correlation between the STP and GC model predictions relative to the LN model prediction (Baba et al., 2004). An equivalence score of 1.0 would indicate perfectly equivalent STP and GC model predictions, and a score of 0 would indicate that the models accounted for completely different response properties. We emphasize that this measure of equivalence is distinct from the measure based on prediction accuracy (Fig. 4*A*). Two models might both improve performance, but they could accomplish that improvement by accounting for different aspects of the neural response. Conversely, two model predictions can be closely matched while not resulting in a significant improvement in prediction accuracy.

For the *n *=* *132 neurons with improvements over the LN model, the equivalence of time-varying predictions had a median of 0.29. This value was relatively low, again indicating weak equivalence between the models. However, the median was significantly greater than that for non-improved neurons (0.17; *p *=* *1.72 × 10^−8^, Mann–Whitney *U* test*^k^*). Thus, there are some similarities between the neural dynamics accounted for by the STP and GC models. As in the comparison of prediction improvements, model estimation error biased measured equivalence to be less than the theoretical maximum of 1.0. To determine the upper bound on equivalence we measured partial correlation between predictions by two fits of the same model using separate halves of the estimation data, as was done for the relative performance comparison above. This within-model control produced a median noise-adjusted partial correlation of 0.68 for the STP model and 0.43 for the GC model. Thus, while the STP and GC models show some degree of equivalence in their time-varying predictions, it is lower than would be expected for fully equivalent models.

### Model estimation noise impacts measures of model equivalence

We performed additional controls for the possibility that the low equivalence suggested by Figure 4*A* resulted from model estimation noise. If noise was a significant factor, then equivalence should be higher for models with better prediction accuracy. If the deviations from the LN model prediction were small and mostly reflected noise (Fig. 3*A*), one would expect weak equivalence using this metric. We therefore defined a measure of effect size for each cell as the mean change in prediction correlation for the STP and GC models relative to the LN model. Neurons for which the more complex models did not improve prediction accuracy over the LN model could have a high equivalence score under the metric used in Figure 4*B*, but would also have a small effect size. If the STP and GC models are equivalent, we would expect most cells with significant improvements over the LN model to only have a large effect size if they also had a high equivalence score. However, we did not discern a clear pattern in the data (Fig. 4*C*). Instead, equivalence and effect size were only weakly correlated (*r *=* *0.22, *p *=* *0.0103*^l^*).

Following this evidence for limited equivalence, we asked whether the combined model’s greater predictive power was merely the result of its flexibility to account for either STP or GC, without any benefit from their combination in individual neurons. If this were the case, we would expect that for an improved cell, the prediction correlation of the combined model should be no greater than the larger of the prediction correlations for the STP and GC models (Fig. 4*D*). Instead, we found that the median prediction correlation was significantly higher for the combined model (median difference 0.0249, Wilcoxon signed-rank test, two-sided, *p *=* *1.41 × 10^−14^*^m^*). This result indicates that simultaneous inclusion of STP and GC mechanisms provides extra explanatory power for some auditory cortical neurons.

**Figure 5.**
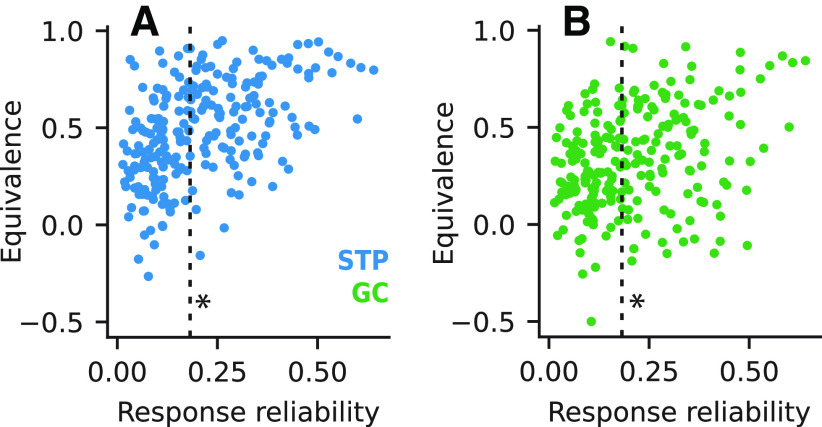
Within-model equivalence ordered by response reliability. ***A***, Scatter plot compares within-model equivalence scores for the STP model compared with response reliability (*n *=* *237 neurons recorded with the larger stimulus set). Dashed line indicates median reliability. Within-model equivalence was significantly higher for neurons with above-median reliability (median high reliability: 0.61, low reliability: 0.36, Mann–Whitney *U* test, *p =* *6.01 × 10^−12^). ***B***, Within-model equivalence scores for the GC model versus reliability, plotted as in ***A***. Again, within-model equivalence was significantly higher for neurons with above-median reliability (median high reliability: 0.42, low reliability: 0.25, *p =* *3.91 × 10^−5^).

We also quantified the extent to which our equivalence analyses were robust to noise in our data. For each neuron included in the within-model equivalence analysis, we compared the reliability of the recorded neural response (Eqs. 8–12) to that neuron’s equivalence score for each of the STP and GC models. For within-model comparisons, we expected to find an association between more reliable neural responses and higher equivalence scores. To determine whether this was the case, we split the neurons into two groups with below-median or above-median reliability (Fig. 5). We found that cells with more reliable neural responses indeed had significantly higher within-model equivalence scores both for the STP model (two-sided Mann–Whitney *U* test, *p *=* *6.01 × 10^−12^*^n^*) and for the GC model (*p *=* *3.91 × 10^−5^°). Notably, the median equivalence scores among more reliable cells (STP: 0.61, GC: 0.42) closely matched the adjusted medians obtained in the previous analysis. We also observed that for the GC model, the maximum equivalence score dropped off considerably for cells with lower reliability.

Finally, we asked whether excluding neurons with low response reliability would have a significant impact on the equivalence results reported above. When we repeated the comparison of relative performance improvements (Fig. 4*A*), we found a substantial effect of excluding the low-reliability data. Relative improvements for the STP and GC models were much more correlated (*r *=* *0.34, *p *=* *1.20 × 10^−7^*^q^*), as were relative improvements for within-model comparisons (STP: *r *=* *0.66, *p *=* *1.18 × 10^−16^*^r^*, GC: *r *=* *0.411, *p *=* *2.61 × 10^−6^*^s^*). For the equivalence of time-varying predictions (Fig. 4*B*), we saw less of a change after excluding low-reliability cells. Median equivalence was comparable to the previous values both for improved neurons (0.31) and for non-improved neurons (0.18), and these values were still significantly different from one another (two-sided Mann–Whitney *U* test, *p *=* *1.23 × 10^−8^*^p^*). These results suggest that the comparison of relative performance improvements is much more sensitive to noise in neural responses than the equivalence score analysis.

### Simulations highlight distinct functional properties of STP and GC models

**Figure 6.**
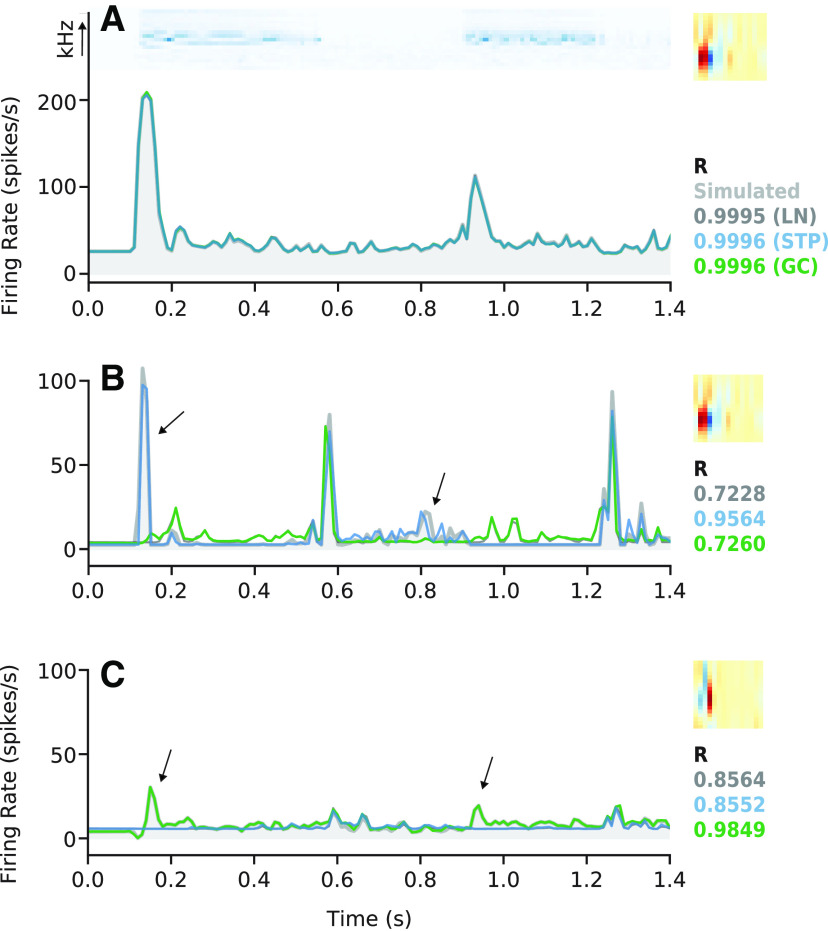
Model performance for simulated data. ***A***, Simulation based on the fitted LN model from Figure 3*A*. Simulated PSTH response to one stimulus (spectrogram at top) based on an LN model is plotted in gray shading. Predicted PSTHs for each model (LN, STP, or GC) are overlaid, and model prediction correlation is indicated at right (LN: dark gray, STP: blue, GC: green). For this linear neuron, all three models perform nearly identically. The linear filter fit using the LN model is shown at the top right. ***B***, Model fits for simulation based on the STP model from Figure 3*B*, plotted as in ***A***. ***C***, Model fits for simulation based on the GC model from Figure 3*C*.

The weak equivalence of the GC and STP models (Fig. 4) suggests that these models account for functionally distinct response properties in A1. As one final control for the possibility that estimation noise could have biased our equivalence results, we analyzed data simulated using the different models. We fit the LN, STP, and GC models to three simulated neural responses (Fig. 6). Each simulation was generated by using a fitted model to predict responses to a set of natural stimuli and treating the model’s prediction as ground-truth for subsequent fitting. Because the simulated responses could be generated noise-free, any difference in STP versus GC model performance could be attributed to differences in their ability to account for nonlinear response properties. The first simulated response was generated using the LN model fit to a cell that showed no improvements for the STP or GC models (Fig. 3*A*). The other simulations were generated using the STP and GC model fits from neurons for which the STP or CG models, respectively, performed better than the LN model (shown in Fig. 3*B*,*C*).

As expected, all three models were able to reproduce the LN simulation nearly perfectly (Pearson’s *R* = 0.9995, *R* = 0.9996, and *R* = 0.9996 for the LN, STP, and GC models, respectively; Fig. 6*A*). However, when fit to the STP simulation, the GC model was no better than the LN model, but the STP model did perform better (*R* = 0.7228, *R* = 0.9564, and *R* = 0.7260; Fig. 6*B*). Conversely, when fit to the GC simulation, the STP model performed no better than the LN model while the GC model did (*R* = 0.8564, *R* = 0.8552, and *R* = 0.9849; Fig. 6*C*). This pattern of different performance confirmed that that the STP and GC models did account for distinct neuronal dynamics.

### Model fit parameters are consistent with previous studies

Since both the STP model and the GC model used in this study were designed to replicate previous studies (Rabinowitz et al., 2012; Lopez Espejo et al., 2019), it is important to verify that the models behaved consistently with these previous observations. This consideration was of particular relevance for the GC model since it had not previously been fit using a natural sound dataset. To test for consistency, we analyzed the distributions of their fitted parameter values for comparison with the previous reports.

**Figure 7.**
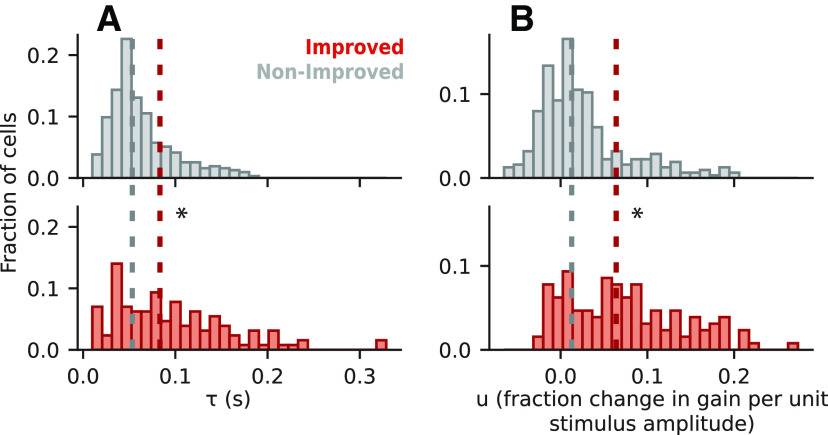
Parameter fit values for the STP model. ***A***, Distribution of *τ*, representing the time constant for the recovery of synaptic vesicles, Top panel shows data for non-improved neurons (gray) and bottom panel for improved neurons (red). Median values for non-improved (0.0533 s) and improved (0.0833 s) neurons were significantly different (*p *=* *3.53 × 10^−6^, two-sided Mann–Whitney *U* test, * *p *<* *0.05), indicating a longer time constant for the improved cells. ***B***, Distribution of *u* values, representing release probability (i.e., the fraction change in gain per unit of stimulus amplitude). Medians for non-improved (0.0128) and improved (0.0641) neurons were significantly different (*p *=* *2.92 × 10^−13^), showing higher release probability for neurons with improved performance over the LN model.

For the STP model (Fig. 7), we found that the median values of both the time constant (*τ*) and fraction gain change (*u*) parameters were significantly higher for improved versus non-improved cells (Mann–Whitney *U* tests, two-sided, *p *=* *3.53 × 10^−6^ and *p *=* *2.92 × 10^−13^, respectively*^t^*^,^*^u^*). The difference was more pronounced for the *u* parameter, and nearly all cells had positive values for the *u* parameter. The large impact of the *u* parameter and predominance of depression over facilitation agreed with published results (Lopez Espejo et al., 2019).

**Figure 8.**
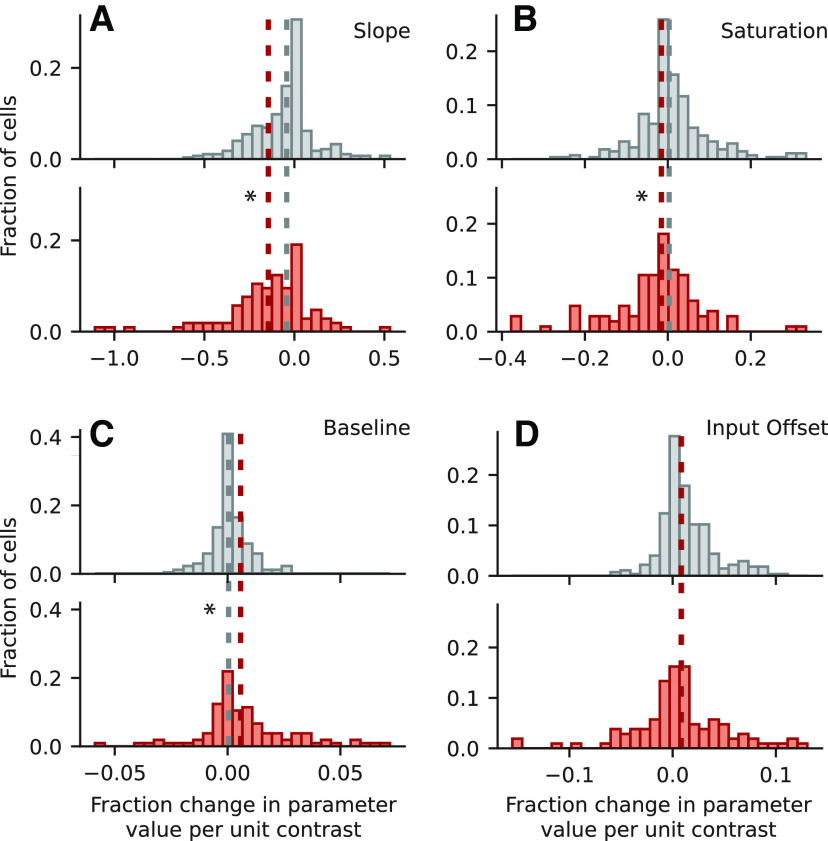
Parameter fit values for the GC model. ***A***, Histogram of effect of contrast on *k*, representing the slope of the output nonlinearity, plotted for non-improved neurons (gray, top) and improved neurons (red, bottom) as in Figure 7. The negative median value for improved (−0.14) cells indicates a decrease in slope during high-contrast conditions. This median was significantly more negative than for non-improved neurons (−0.042, *p *=* *1.22 × 10^−4^, two-sided Mann–Whitney *U* test). Asterisk indicates *p *<* *0.05. ***B***, Histogram of contrast effect on *a* (saturation level), plotted as in ***A***. Median values for non-improved (0.0031) and improved (−0.0156) neurons were significantly different, indicating a decreased response amplitude in high contrast conditions (*p *=* *8.01 × 10^−6^). ***C***, Histograms of contrast effect on *b* (baseline of the output nonlinearity). Medians for non-improved (0.0005) and improved (0.0058) neurons were significantly different, indicating an increase in baseline for high contrast (*p *=* *5.90 × 10^−7^). ***D***, Distribution of contrast effect on *s* (input offset). There was no significant difference between medians for non-improved (0.0088) and improved (0.0082) neurons (*p *=* *0.85).

For the GC model (Fig. 8), the impact of contrast on the slope (*k*) parameter of the output nonlinearity was significantly more negative for improved neurons than for non-improved neurons (Mann–Whitney *U* tests, two-sided; *p *=* *1.22 × 10^−4^*^v^*). This indicates a decrease in neural gain during high contrast sounds, which is consistent with models fit using dynamic random chord (RC-DRC) stimuli (Rabinowitz et al., 2012). Additionally, compared with non-improved cells, the relationship between contrast and saturation (*a*) was significantly more negative, and the relationship between contrast and baseline (*b*) was significantly more positive for improved cells (*p *=* *8.01 × 10^−6^ and *p *=* *5.90 × 10^−7^, respectively*^w^*^,^*^x^*). However, we observed no significant difference in the effect of contrast for the input offset (*s*) parameter, which did change for RC-DRC stimuli (*p *= 0.852 y). As observed in the previous study, the net result of increasing contrast was to decrease the gain of neural responses, and thus the overall effects of changing contrast on response gain were consistent between studies.

### Relationship between baseline neural properties and model performance

**Figure 9.**
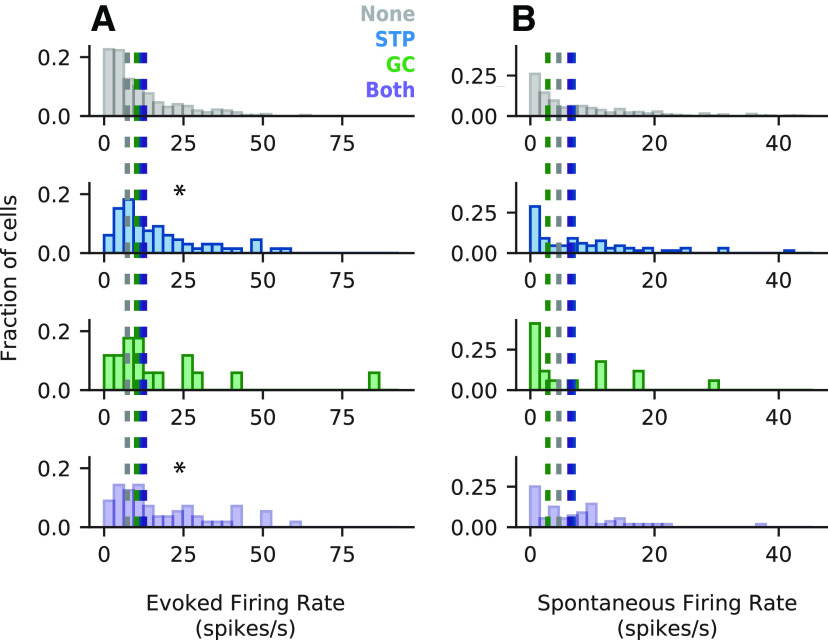
Mean evoked and spontaneous firing rates grouped by nonlinear model performance. ***A***, Histograms of mean evoked firing rate for four mutually exclusive groups of neurons: no significant improvement in prediction accuracy for the nonlinear models (gray, top), significant improvement for the STP model relative to the LN model (blue, middle-top), significant improvement for the GC model relative to the LN model (green, middle-bottom), or significant improvement for both the STP and GC models or the GC+STP model (purple, bottom). Median evoked firing rate was significantly higher for the STP (11.7 spikes/s) and Both (12.7 spikes/s) groups than for the None group (7.31 spikes/s, Mann–Whitney *U* test, *p =* *1.68 × 10^−3^ and *p =* *2.67 × 10^−3^, respectively, adjusted for multiple comparisons). All other comparisons were not statistically significant (*p* > 0.05). ***B***, Histograms of spontaneous firing rate for each model, plotted as in ***A***. None of the groups was significantly different from the others (*p* > 0.05).

Previous work has reported that spontaneous firing rates are related to the predictive power of the STP model (David and Shamma, 2013). We therefore asked whether there was a basic functional property that could predict whether a particular neuron would benefit from one nonlinear model or another. While we could not distinguish neuronal cell type (e.g., excitatory versus inhibitory neurons), we could measure basic aspects of spiking activity, namely spontaneous and evoked firing rates, that might correspond to biological properties of the neurons. To this end, we split the neurons into four mutually exclusive groups. The first three consisted of: neurons for which none of the nonlinear models significantly improved prediction accuracy (None, *n *=* *327), neurons for which the STP model significantly improved prediction accuracy (STP, *n *=* *66), and neurons for which the GC model significantly improved prediction accuracy (GC, *n *=* *17), respectively. The fourth group contained neurons for which both the STP and GC models significantly improved prediction accuracy or for which the GC + STP model alone significantly improved prediction accuracy (Both, *n *=* *56). We then separately compared the median evoked and spontaneous firing rates between each group (Fig. 9).

Of the 12 comparisons made, most (10/12) were not statistically significant ($p^{*}>0.05^{z-ii}$). Only two showed a significant difference: median evoked firing rate was significantly higher for the STP (11.7 spikes/s) and Both (12.7 spikes/s) groups compared with the None (7.31 spikes/s) group (Mann–Whitney *U* test, p* = 1.68 × 10^−3^ and p* = 2.87 × 10^−3^, respectively*^jj^*^,^*^kk^*, adjusted for multiple comparisons). Thus, there was a small correlation between STP effects and the magnitude of evoked activity.

### Greater relative contribution of contrast gain to encoding of noisy natural sounds

**Figure 10.**
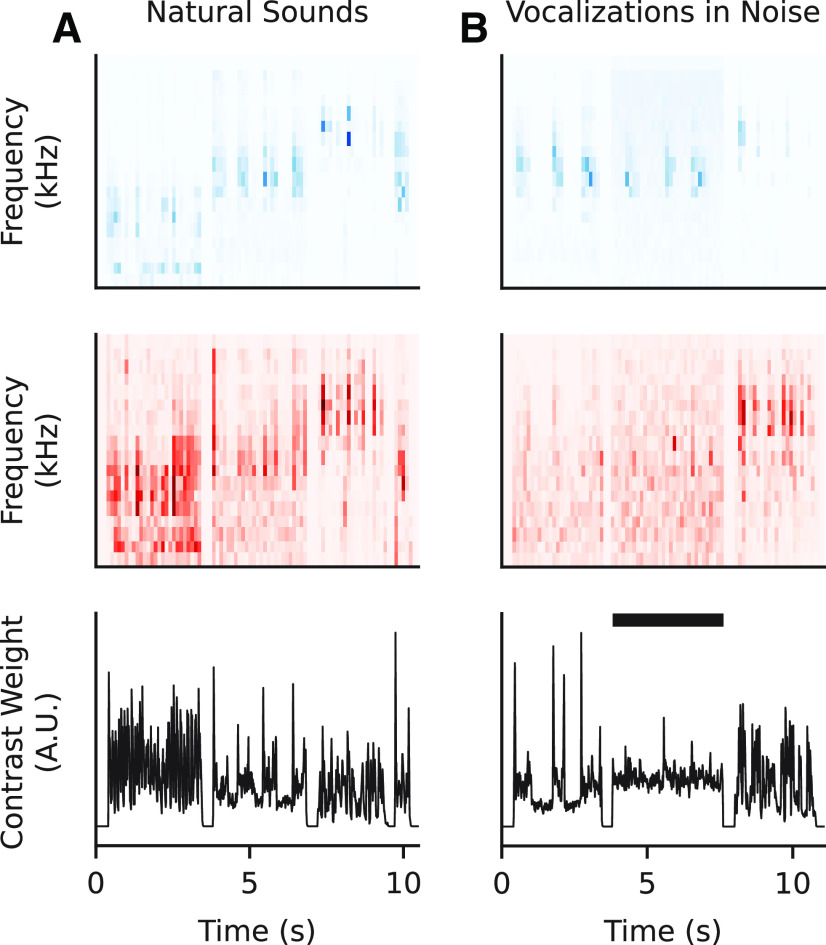
Comparison of natural sound and clean/noisy vocalization properties. ***A***, Top to bottom, stimulus spectrogram (blue), contrast (red), and frequency-summed contrast (black) for a sequence of three natural sound samples. ***B***, Same as in ***A***, but for vocalizations. The vocalization set contained interleaved trials of ferret vocalizations with and without additive noise. Black bar indicates segment with noise added.

**Figure 11.**
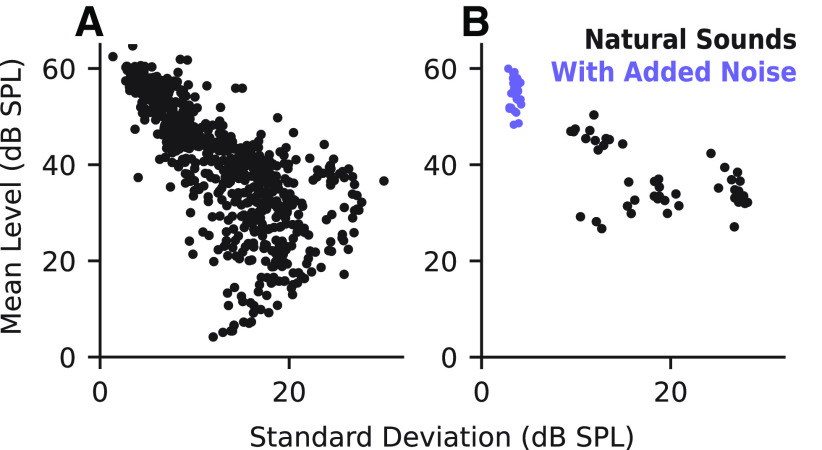
Comparison of contrast properties between stimulus sets. ***A***, Scatter plot of SD and mean level (dB SPL) for each natural sound spectrogram. The distribution indicates a smooth variation in contrast level. ***B***, Comparison of SD and mean for clean/noisy vocalizations. There is a clear grouping of noisy (low-contrast) and clean (high-contrast) stimuli.

We also compared performance of the STP and GC models on a smaller dataset collected with clean and noisy ferret vocalizations (Fig. 10). Previous studies using stimulus reconstruction methods argued that both STP and GC are necessary for robust encoding of noisy natural signals (Rabinowitz et al., 2013; Mesgarani et al., 2014). The inclusion of additive noise should reduce contrast by increasing the mean sound energy and reducing variance. We compared the mean and SD of each sample for the two datasets to see whether there were any systematic differences in contrast. We found that the natural sounds dataset smoothly spanned the range of observed means and SDs. Meanwhile, the vocalization dataset formed two distinct categories: a high-contrast group of clean vocalizations and a low-contrast group of noisy vocalizations (Fig. 11). A small set of the natural sounds overlapped with the noisy vocalizations because they had similar noise characteristics.

**Figure 12.**
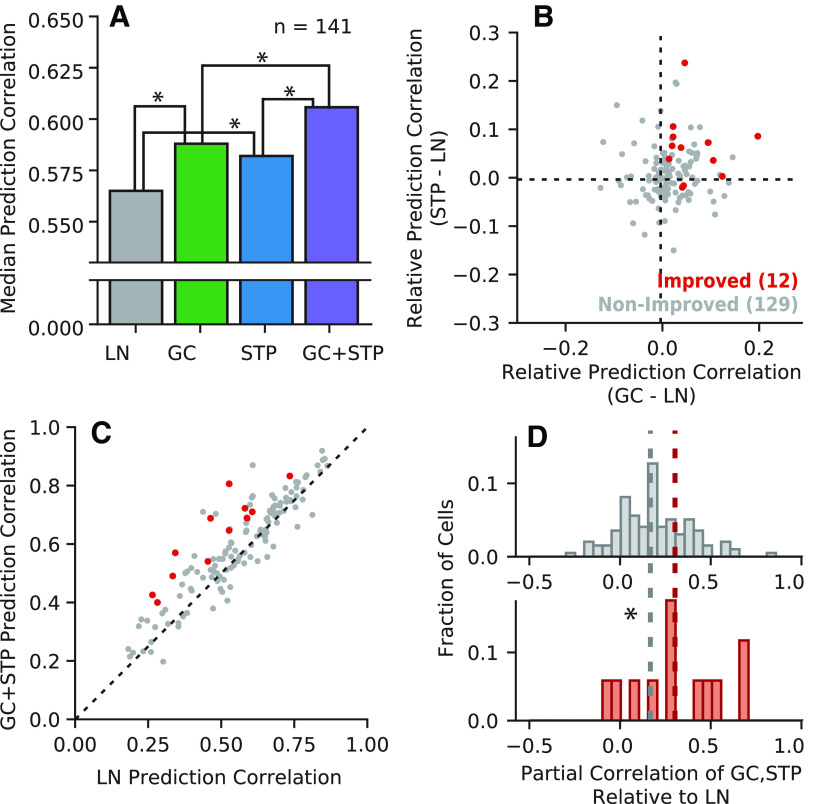
Comparison of model performance for data including clean and noisy vocalizations. ***A***, Median prediction correlation (*n *=* *141) for each model. Statistical significance of median differences was determined via two-sided Wilcoxon signed-rank tests (*p <* *0.05). Unlike the natural sound data (Figure 2), performance was not significantly different between the GC and STP models. ***B***, Change in prediction correlation for the GC (horizontal axis) and STP (vertical axis) models relative to the LN model for each neuron (*r *=* *0.18, *p *=* *3.45 × 10^−2^). ***C***, Prediction correlations for the LN model compared with the combined model for each neuron, grouped by whether the combined model showed a significant improvement (red, *n *=* *12, *p *<* *0.05, permutation test) or not (gray, *n *=* *129). ***D***, Histogram of equivalence for non-improved (top, gray) and improved neurons (bottom, red). Median equivalence for improved cells (0.30) was significantly greater than for non-improved cells (0.16, Mann–Whitney *U* test, *p =* *0.0449).

We compared model performance and equivalence, using the same approach as for the natural sound data above (Fig. 12). For the noisy vocalization data, we again found that both the STP and GC models performed significantly better than the LN model and that the combined model performed significantly better than either the STP or GC model individually (Wilcoxon signed-rank tests, two-sided, *p *=* *0.0058, $p=1.70\times 10^{-8},\;p=3.69\times 10^{-9}$, and $p=4.27\times 10^{-5}$, respectively*^ll^*^,^*^mm^*^,^*^nn^*^,^*^oo^*). However, unlike for the natural sound data, performance of the STP and GC models themselves was not significantly different (Wilcoxon signed-rank test, two-sided, *p *=* *0.105*^pp^*). This difference indicates a relative increase in the performance of the GC model when applied to noisy vocalizations. This effect is consistent with the hypothesis that gain control plays a bigger role in shaping neural responses for stimuli with large fluctuations between high and low contrast.

The equivalence analysis also produced similar results for the noisy vocalizations data. The change in prediction correlation was only weakly correlated for the STP and GC models (*r *=* *0.18, *p *=* *3.5 × 10^−2^*^qq^*). Moreover, partial correlation between STP and GC predicted responses was modest for improved cells (median 0.30), but significantly higher than the median for non-improved cells (median 0.16, Mann–Whitney *U* test, two-sided, *p *=* *0.0449*^rr^*). However, the small number of significantly improved cells in this dataset (*n *=* *12/141) made drawing definitive conclusions difficult. This smaller set of improved cells likely reflects the fact that the amount of estimation data was smaller for the noisy vocalizations than for the clean natural sounds.

## Discussion

We found that encoding models incorporating either GC or STP explained complementary aspects of natural sound coding by neurons in A1. Although we observed some degree of equivalence between models, the overlap was modest relative to what would be expected if both models explained the same functional properties (Fig. 4). Instead, a novel model that incorporated both STP and GC mechanisms showed improved performance over either separate model (Fig. 2). It is well established that the LN model fails to account for important neural activity in real-world, natural stimulus contexts (Theunissen et al., 2000; Machens et al., 2004). This work supports the idea that both forward adaptation, as might be mediated by STP, and feedback inhibition, as might be mediated by GC, play a role in these contextual processes.

### Assessing equivalence of encoding models

A major goal of this study was to establish a framework for systematically comparing the functional equivalence of complex encoding models. The STP and GC models served as a useful case study for analysis of equivalence: both models emulate adaptation following sustained stimulation, and it is not immediately obvious whether they account for distinct contextual effects. By comparing the performance of these alternative models on the same natural sound dataset, we were able to determine that they in fact account for distinct functional properties. We explored two methods for assessing model equivalence: the degree to which two alternative models improve prediction accuracy by the same magnitude for the same neuron, and the degree to which the time varying responses predicted by those models are identical. Accurate assessment using either equivalence metric requires accounting for noise in model parameter estimates, which can bias equivalence metrics toward lower values. In the current study, we found that the latter metric based on the partial correlation between predicted time-varying responses was more robust to noise and thus may be a more reliable measure of equivalence.

Many other encoding models have been developed previously, each representing a separate hypothesis about nonlinear dynamics in auditory encoding (Schinkel-Bielefeld et al., 2012; Kozlov and Gentner, 2016; Williamson et al., 2016; Harper et al., 2016; Willmore et al., 2016). In some cases, the equivalence of encoding models can be established analytically (Williamson et al., 2015). However, with the development of convolutional neural networks and related machine learning-based models, future models are likely to only become more complex and difficult to characterize (Kell et al., 2018; Keshishian et al., 2019). Alternative models cannot be compared easily because of the many experimental differences between the studies in which they are developed. Performance depends not only on the model architecture itself, but also on numerous details of the fitting algorithm and priors that themselves are optimized to the specific dataset used in a study (Thorson et al., 2015). This complication leaves an important question unanswered: if some number of these models all improve predictive power, does each model’s improvement represent unique progress in understanding the function of auditory neurons or is there overlap in the benefits that each model provides? The equivalence analysis described in this study provides the necessary tools to begin answering this question.

### Increased impact of gain control for stimuli in acoustic noise

The greater relative performance of the GC model for the dataset including noisy vocalizations indicates a behaviorally-relevant regime in which gain control contributes significantly to neural coding in noisy acoustic conditions. The fact that clean and noisy stimuli naturally cluster into high-contrast and low-contrast groups, respectively, may explain the GC model’s increased impact. As a result of this division, a combination of clean and noisy stimuli provides a naturalistic replication of the switches between high-contrast and low-contrast contexts that was used with RC-DRC stimuli in other studies (Rabinowitz et al., 2012; Lohse et al., 2020). In comparison, these binary switches between contrast regimes were absent from the dataset containing natural sounds without added noise, which instead smoothly spanned the contrast space.

In contrast to the variable GC model performance, the consistent performance of the STP model across both datasets suggests that STP operates across a wider range of stimuli and is relevant to sound encoding even in the absence of acoustic noise. Previous studies have shown that some nonlinear computations are required in addition to the LN model to account for noise-invariant neural responses in auditory cortex (Moore et al., 2013; Rabinowitz et al., 2013; Mesgarani et al., 2014). The complementary effects of gain control and STP reported here are consistent with the idea that both mechanisms contribute to robust encoding of noisy auditory stimuli.

### Comparison with previous studies of gain control

In order to adapt the GC model to analysis of natural sound data, we made some important changes to the original implementation (Rabinowitz et al., 2012). First, although Rabinowitz and colleagues imposed the contrast profiles of their stimuli by design, natural stimuli contain dynamic fluctuations in contrast with no predetermined window for calculating contrast. As a result, we were required to make decisions about parameters governing the contrast metric: the spectral and temporal extent of the window used to calculate contrast and the temporal offset needed to emulate the dynamics with which contrast effects are fed back to the response.

We used a 70-ms, spectrally narrowband convolution window with a 20-ms temporal offset, which worked best on average in our initial analysis. However, there was variability in the best window for different cells, so model performance may be further improved if these parameters are optimized on a cell-by-cell basis. Optimal plasticity parameters also varied substantially between cells for the STP model. Variability in contrast integration windows may reflect similar biological differences in how cells adapt to contrast. For example, a recent study reported that the timescale of the impacts of contrast on neural gain varied among neurons both in auditory cortex and in two subcortical regions (Lohse et al., 2020).

A second important difference from the original GC model is that we were not able to differentiate high contrast sounds with high SD from those with an exceptionally low mean level since both cases can result in a large coefficient of variation. In the original study, Rabinowitz et al. (2012) were able to fix mean level across stimuli to avoid this potential confound. Despite these differences, our results broadly replicated the original findings.

### Mechanisms mediating effects of sensory context on auditory cortical responses

Previous work has described cortical layer 6 neurons as a mechanistic source of gain control in the visual system (Olsen et al., 2012). Other adaptive mechanisms like SSA have also been shown to arise from cortical circuits (Natan et al., 2015). However, in the auditory system gain control has been attributed both to cortical feedback and to feedforward adaptation arising in subcortical regions (Lohse et al., 2020). In this case, STP could contribute to the feedforward process (Rabinowitz et al., 2011).

In the current study, the GC model was formulated as a feedback mechanism (Rabinowitz et al., 2012), while the STP model described a feedforward mechanism (David and Shamma, 2013). Although we did not directly measure circuit properties, we found that a model combining both mechanisms provided the most accurate predictions overall. Thus, our results are consistent with the hypothesis that both contribute to context-related processing in A1. Future experiments involving direct manipulations of synaptic plasticity and/or inhibitory feedback mechanisms can provide explicit insight into the mechanisms underlying these functions.

## Synthesis

Reviewing Editor: Miriam Goodman, Stanford University Decisions are customarily a result of the Reviewing Editor and the peer reviewers coming together and discussing their recommendations until a consensus is reached. When revisions are invited, a fact-based synthesis statement explaining their decision and outlining what is needed to prepare a revision will be listed below. The following reviewer(s) agreed to reveal their identity: Ben Willmore, Jan Clemens.

Your manuscript has been evaluated by two reviewers who conferred in a post-review on-line discussion. The consensus view is that the study addresses a very interesting question - whether synaptic plasticity (STP) and contrast gain control (GC) are complementary mechanisms operating in neurons in auditory cortex, or whether they are overlapping descriptions of the same phenomena. The principal observation of computational non-equivalence of STP and GC is largely expected given the difference in their formulation and in their position in the LN (before or after the filter). This expectation does not undermine the contribution of the study, however, as it remains important to show that that this non-equivalence is of practical importance - that signatures of both types of mechanisms can be found in “real” neurons. The results of this study are therefore of potential interest to a wide range of researchers who want to model adaptation in auditory and other sensory systems. The reviewer consensus is to improve the communication of limitations of the study and of the efforts to compare the predictive value of the types of models being studied. In particular, clarity would be improved by explicitly noting potential limitations due to over-fitting or estimation methods being compromised by noise in the data. Their specific point-by-point critiques are included in this report to assist you in revising the manuscript while keeping in mind the overarching recommendation to assist readers in deciphering both the benefits and limitations of the study.

Reviewer 1 (minor points for improvement)

Minor points

pg. 20 “neurons for which the STP model significantly improved prediction accuracy (“GC", n = 66), and neurons for which the GC model significantly improved prediction accuracy (“STP", n = 17), respectively”. I think the labels of the GC and STP groups have been switched.

pg. 20 “We also compared performance of the STP and GC models on a smaller data set collected with clean and noisy ferret vocalizations 9”. I’m not sure what the “9” is doing here - maybe a reference to Figure 9?

Figure 10 legend. “Comparison of standard deviation and mean for clean/noisy vocalizations There” Missing “.”

There are a few typos in the references:

* Moore et al “Cortex:Hearing” is missing a space

* Sadagopan et al “UnderlyingSelectivity” is missing a space

* Sahani & Linden missing volume information

Reviewer 2 (major and minor points for improvement)

General major points:

1. Some of the analyses seem unnecessarily complicated. Having to understand the different control analyses and multiple levels of correction for finite sample sizes distracts from the main argument and makes the paper hard to read and unnecessarily long. For instance, model performance and the pattern of predictions are analysed separately: The authors first show that different adaptation models improve the model performance in different ways (Fig. 4A) and then show that LN+STP and LN+GC models produce different response patterns. The latter is done by calculating the partial correlation between LN+STP and LN+GC, accounting for common correlations with the LN model (Fig. 4B,C). Wouldn’t it be simpler to combine both analyses: Calculate the partial correlation between the full LN+STP+GC model and the *response* (not the prediction) accounting for the LN+STP or the LN+GC correlation? This would quantify the unique contribution of GC or of STP to the model performance in a single analysis step. This unique contribution would have to arise from differences in the patterns of predictions.

2. To account for estimation noise, *all* analyses should be done on splits of the data. Currently, only the within-model controls, but not the between-model comparisons are performed on data splits. For instance, in the comparison of performance improvements (p12, last paragraph), the authors compare the model improvement for the STP and GC models fitted to the full data and find a low correlation of 0.18. They then estimate a base-line by splitting their data in half and comparing the within-model improvement for the two models fitted on each half of the data. To put the between and within model analyses on the same footing, they should both be done on halves of the data. The same applies for the partial correlation analyses.

3. To correct finite data sizes in the equivalence analysis a *global* scaling factor is used. However, different cells may be affected by the reduction of the training data in different ways and a global scaling factor seems inappropriate in the presence of this potential heterogeneity. Given the method they describe, it is unclear why the authors us a global scaling factor (based on a median) instead of correcting partial correlations with a scaling factor estimated for each cell.

Specific major points:

4. p12, last paragraph: The correlation in the improvement with a model fitted to different halves of the data is very low (0.5 and 0.3). Is this because the improvements are small and therefore noisy? How reproducible are the performance values (“prediction correlations”) estimated from fits to different halves of the data? Does this low correlation indicate high estimation noise (unreliable convergence of the fitting algorithm, multiple local minima in MSE) or noisy/non-stationary data?

5. I find the analysis described in Fig. 4D unnecessarily complicated. The authors’ aim here is to test “whether the combined model’s greater predictive power was merely the result of its ability to simply account for either STP or GC, without any benefit from their combination in individual neurons”. I believe this analysis only makes sense for the improved cells - cells for which the LN+STP+GC model outperforms that of the LN model. By performing this analysis for *all* cells, the authors are forced to make confusing statements: For instance, the authors state: “However, a majority of the non-improved neurons appeared to utilize only one mechanism or the other (189/336, or 56%)” (p16, top).

1. Doesn’t “non-improved” mean that inclusion of STP+GC does not improve performance over LN alone (p12, top)? In other words, that these cells employ neither STP nor GC? But it looks as if for 44% of these cells, the STP+GC model outperforms the best of the STP or the GC models. These means these cells must employ at least one of the two adaptation mechanisms. Shouldn’t these cells be called “improved” then? Using two different comparisons (LN vs STP+GC and max(STP, GC) vs STP+GC) leads to two different, and partially contradictory, notions of “improved” - probably due to estimation noise.

2. Is this surprising? Wouldn’t one expect a 50:50 split if both values were identical plus some noise? Restricting the analysis to the valid subset prevent the authors from making these statements. In addition, a statistical test should be employed to identify the subset of “non-improved” cells that utilize only one or both mechanisms.

Minor points:

1. The STP+GC model is worse than the best of the STP-only or GC-models model (Fig. 4D). Theoretically, the STP+GC should never perform worse than either of the simpler models even if the data is noisy. If this stems from overfitting then it needs to be stated.

2. Predictions from LN, STP and GC models are shown in Fig. 3 and 5. Predictions from the full STP+GC model are never shown. Such an example could be used to further illustrate the complementarity of the STP and the GC model.

3. The simulation results a convincing. Could a simulation approach be used to show the effect of noisy data on their analysis? For instance, models with different levels of noise added to the data could be fitted, to show at what noise level their procedure looses its ability to differentiate between the different model types.

4. top of p16: “prediction accuracy was greater for the combined model than for either the STP or GC model individually for 108 of the 132 improved cells (0.82%)”. “0.82” should be “82%”.

5. typos on p20: end of first paragraph: “...between each group 8.”, first sentence in last paragraph: “... noisy ferret vocalizations 9.”
